# Higher comorbidity burden is associated with lower self-reported quality of life after stroke

**DOI:** 10.3389/fneur.2022.1023271

**Published:** 2022-11-10

**Authors:** Marlene Heinze, Lisa Lebherz, David Leander Rimmele, Marc Frese, Märit Jensen, Ewgenia Barow, Iris Lettow, Levente Kriston, Christian Gerloff, Martin Härter, Götz Thomalla

**Affiliations:** ^1^Department of Neurology, University Medical Center Hamburg-Eppendorf, Hamburg, Germany; ^2^Department of Medical Psychology, University Medical Center Hamburg-Eppendorf, Hamburg, Germany; ^3^Quality Management and Clinical Process Management, University Medical Center Hamburg-Eppendorf, Hamburg, Germany

**Keywords:** comorbidity burden, polypharmacy, health-related quality of life, stroke, CCI

## Abstract

**Introduction:**

This study assesses the association of comorbidity burden and polypharmacy with self-reported quality of life after stroke.

**Patients and methods:**

We performed a *post-hoc* analysis of a prospective, single-center, observational study of outcome evaluation by patient-reported outcome measures in stroke clinical practice. Consecutive patients with acute ischemic stroke (AIS) were enrolled and self-reported health–related quality of life (HrQoL) was assessed 90 days after acute stroke using the Patient-reported Outcomes Measurement Information System 10-Question Short-Form (PROMIS-10). Comorbidities at baseline were assessed by the Charlson Comorbidity Index (CCI). Polypharmacy was defined as medication intake of ≥5 at baseline. We used linear regression analysis to study the association of CCI, polypharmacy and other clinical covariates with HrQoL after stroke.

**Results:**

Of 781 patients (median age 76 years, 48.4% female) enrolled, 30.2% had a CCI Score ≥2, and 31.5% presented with polypharmacy. At follow up, 71 (9.1%) had died. In 409 (52.4%) reached for outcome evaluation, Global Physical Health T-Score was 43.8 ± 10 and Global Mental Health T-Score was 43.5 ± 8.76, indicating lower HrQoL than the average population. A CCI Score ≥2, higher NIHSS Score, female sex, dependency on others for dressing, toileting and mobility before index stroke, atrial fibrillation and hypertension were independent predictors of worse physical and mental health outcomes, while polypharmacy was not.

**Conclusion:**

In patients with AIS, high comorbidity burden and polypharmacy are frequent. Comorbidity burden at admission is independently associated with worse self-reported physical and mental health three months after stroke.

## Introduction

Establishing predictive factors of health-related quality of life (HrQoL) after stroke is vitally important to ensure optimal post-stroke care. Index stroke severity (1, 2), bad functional outcome (3), female sex (4), and higher age (5) have been shown to have a detrimental effect on HrQoL after stroke. A high comorbidity burden at time of stroke has recently moved more to the center of interest as an independent predictor of mortality and functional outcome. Comorbidity burden was associated with higher in-hospital mortality (6, 7), length of hospital stay (8), and adverse clinical outcomes (9–11) in stroke patients, but very little data are available on the influence of high comorbidity load on HrQoL. Comorbidity burden is closely linked to polypharmacy, which is typically defined as an intake of ≥5 medications (12) and is considered a growing challenge in clinical practice (13). Stroke is associated with multiple cardiovascular risk factors and comorbidities (e.g., arterial hypertension, diabetes and atrial fibrillation) and preventative medical treatment of these conditions contributes to polypharmacy (14, 15). Polypharmacy has in turn been associated with lower HrQoL (16), an increased frequency of hospitalization, length of hospital stay, in-hospital mortality, disability and adverse drug reactions in population-based studies (17). Detrimental effects of stroke in domains of both physical and mental health are not well-represented in commonly used clinical rating and outcome scales (18), but can severely diminish quality of life (19). The effect of stroke on HrQoL can be assessed with the use of patient-reported outcome measures (PROMs), which have become increasingly important for outcome evaluation with regards to value-based health care (20, 21). A previous, ongoing study of outcome evaluation by PROMs in stroke clinical practice (EPOS) assessed the implementation of “The International Consortium for Health Outcome measurement Standard Set for Stroke (ICHOM-SSS)” in acute stroke care and identified impairments in physical and mental health across multiple domain (22). The EPOS study was registered at ClinicalTrials.gov, NCT03795948.

Data on the influence of comorbidity burden and polypharmacy on HrQoL after stroke are scarce. Here, we analyzed the prospective observational EPOS study with the aim to determine the strength of association of a high comorbidity burden and polypharmacy with self-reported quality of life after stroke in mental and physical health domains.

## Patients and methods

### Study design

For this exploratory secondary analysis, we reviewed data on comorbidities and polypharmacy at time of admission for patients enrolled in the EPOS study (23). EPOS is a prospective exploratory observational and implementation study with a longitudinal design to evaluate patient-reported outcome measures of physical and mental health after acute ischemic stroke (AIS), intracranial hemorrhage and transitory ischemic attack in clinical practice. For the current analysis, we only included patients with diagnosis of AIS confirmed by brain imaging or comprehensive clinical evaluation. All patients or legal representatives provided informed consent. Informed consent was waived for patients who died before consent could be obtained. The study protocol was approved by the ethics committee of the Hamburg chamber of physicians.

### Baseline data

We evaluated clinical and demographic data recorded at baseline and during treatment at the stroke unit of our hospital according to the International Consortium for Health Outcome measurement Standard Set for Stroke (24). Stroke severity was assessed using the National Institutes of Health Stroke Scale (NIHSS). Information on comorbidities and polypharmacy were collected from electronic medical records. Pre-existing comorbidities were assessed according to the Charlson Comorbidity Index (CCI) (25). The CCI is an extensively validated index comprising 19 diseases that are weighted according to their association with mortality and can thus be summarized. We used the modified CCI, which has been validated in ischemic stroke patients and excludes hemiplegia and previous cerebrovascular events (10). We further recorded atrial fibrillation, arterial hypertension, and hyperlipidemia, which are considered to be frequent and important comorbidities in stroke but are not captured by the CCI. Atrial fibrillation was considered to be present if either reported in the patients' history or detected on admission or during monitoring at the stroke unit. Polypharmacy was defined as the intake of ≥5 medications at time of stroke onset. Medication was grouped into antihypertensives, antiplatelet, anticoagulants, antidiabetics, statins, antidepressants/antipsychotics and others.

### Outcome assessments

Outcome was assessed at 90 days after stroke according to the International Consortium for Health Outcome measurement Standard Set for Stroke, including information on survival and patient-reported physical and mental health status. The PROMIS 10-Question Short Form (PROMIS-10) was administered as a pen-and-paper questionnaire. PROMIS 10 derives T-scores for the domains “Physical Health” and “Mental Health” from 4 questionnaire items each. The general population reference norm is a T-Score of 50 with a SD of 10 with lower values indicating a poorer outcome. Two further single-question items address “General Health” and “Social Functioning” with a score reaching from 1 (“poor”) to 5 (“excellent”). The PROMIS-10 has been shown to be a reliable and valid measure of HrQoL in stroke patients (26).

### Statistical analysis

We provide descriptive statistics for all patients included in the analysis. We dichotomized the CCI according to prevalence of comorbidities with a cutoff of ≥2 points defining relevant comorbidity burden. Baseline characteristics were compared between patients with and without relevant comorbidity and between patients with and without polypharmacy. We performed a group comparison of baseline characteristics between patients with available follow-up and those lost to follow-up. To study whether comorbidity burden and polypharmacy are associated with patient-reported outcome measures, we first performed univariate analysis with PROMIS-10 physical and mental health as outcome parameters including the dichotomized CCI, polypharmacy and baseline demographic data and cardiovascular risk factors as independent parameters. Due to high correlation between “Living at home without support” and “Independence in dressing/toileting/mobility” we only included independence. Secondly, we fitted linear regression models including parameters which significantly predicted outcomes with *p* < 0.05 in univariate analysis. All tests were carried out with a two-sided alpha level of 5%. We did not correct for multiple testing seeing as this analysis was considered exploratory. As sensitivity analysis, we repeated the regression analysis with the intention-to-treat population after multiple imputation of missing outcome values based on available baseline data.

## Results

### Patient characteristics at baseline, frequency of comorbidities and polypharmacy

**Figure 1:**
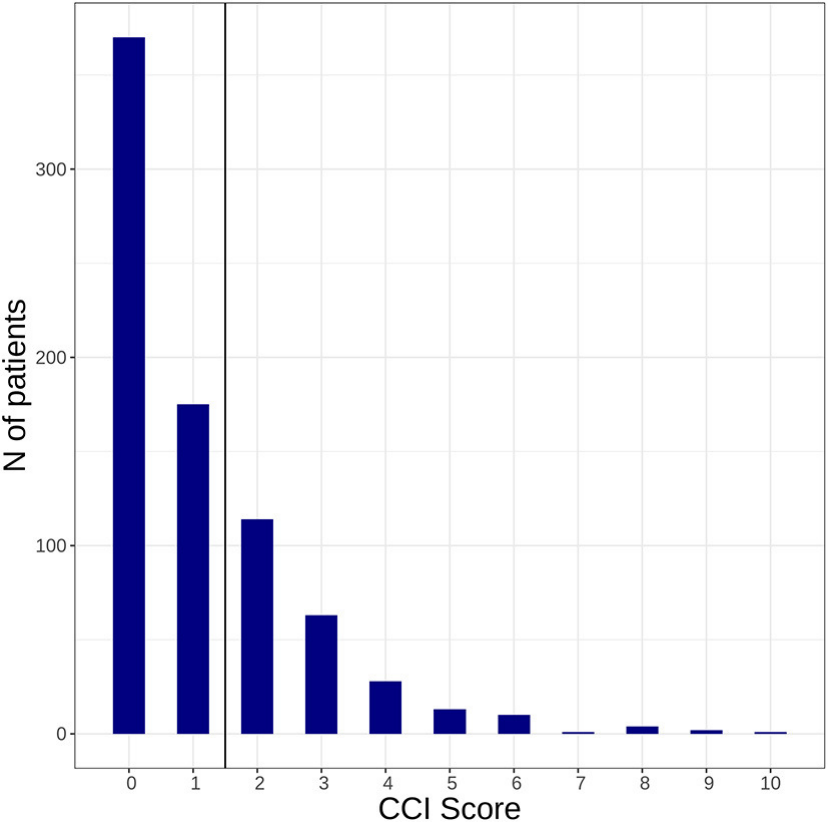
Illustrates how many patients with acute ischemic stroke included in this study presented with a Charlson Comorbidity Index ≥ 2 at baseline.

**Figure 2:**
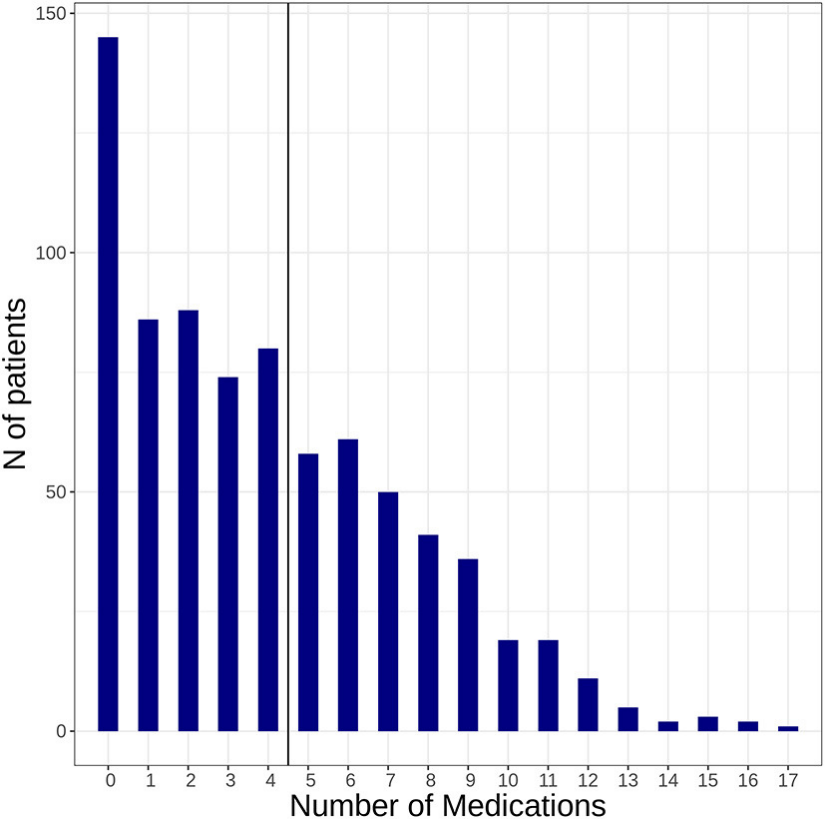
Illustrates how many patients presented with a medication intake ≥ 5 at time of index stroke (baseline).

We included 781 patients with confirmed diagnosis of AIS. Median age was 76 years, 48.4% were female (see Table 1). Patients presented with a median NIHSS of 4 at baseline. Relevant comorbidities defined by a CCI ≥2 were present in 236 (30.2%) patients. The distribution of the CCI score at baseline is shown in Figure 1. Supplementary Figure 1 shows the proportion of patients with each condition included in the CCI. Polypharmacy was present in *n* = 246 (31.5%) of patients at time of index stroke. For numbers of patients taking individual drugs and distribution of medication intake see Figure 2 and Supplementary Figure 2.

**Table 1:** Patient characteristics at baseline.

**Variables**	***N*** = 781^*^
Female [*N*(%)]	378 (48.4%)
Age (Median [*IQR*])	76 [67;83]
CCI score ≥2	236 (30.2%)
CCI score (Median [*IQR*])	1 [0;2]
Polypharmacy [*N*(%)]	246 (31.5%)
Number of medications per patient (Median [*IQR*])	3 [1;6]
NIHSS (Median [*IQR*])	4 [1;10]
Living at home† [*N*(%)]	617 (79.0%)
Independence‡ [*N*(%)]	702 (89.9%)
Atrial Fibrillation [*N*(%)]	239 (30.6%)
Hypertension *N*(%))	466/774 (60.2%)
Hyperlipidemia [*N*(%)]	91/754 (12.1%)

* In case of missing data, N of available for data is displayed † without external support or care ‡ in dressing, mobility and toileting before index stroke.

### Outcome measures and PROMIS-10 scores at 90 days after stroke

Ninety days after stroke, 71 patients had died (9.1%), and 409 (52.4%) patients were reached to obtain PROMIS-10 scores (see Supplementary Table 1). Mean T-Score for Global Physical Health was 43.8 ± 10 and 43.5 ± 8.76 for Global Mental Health. The differences between patients available for follow-up vs. those lost to follow-up are displayed in Supplementary Figure 2.

### Group comparisons by comorbidity burden and polypharmacy

Baseline group comparisons show that patients with high comorbidity burden or polypharmacy respectively were older, less frequently independent in their living situation and everyday life, presented with a higher NIHSS and had higher mortality at 90 days than their respective counterparts. For detailed data please refer to Table 2. Both patient groups reported significantly worse outcomes on all measures of HrQoL when compared to patients with fewer comorbidities and patients without polypharmacy (see Table 2).

**Table 2:** Patient characteristics at baseline compared between patients with/without CCI >2 and with/without polypharmacy.

**Variables**	**CCI** ≥**2**		**CCI**<**2**		***p*** **-Value**	**Polypharmacy**		**No polypharmacy**		***p*** **-Value**
	***N*** **= 236 (30.2%)**		***N*** **= 545 (69.8%)**			***N*** **= 246 (31.5%)**		***N*** **= 535 (68.5%)**		
Female [*N*(%)]	138	(48.4%)	240	(48.4%)	1.000	122	(49.6%)	256	(47.9%)	0.707
Age (Mean ± SD)	77.2	[±10.8]	72.2	[±13.7]	< .001	77.6	[±10.7]	72.5	[±13.5]	< 0.001
CCI Score ≥ 2 [*N*(%)]	-	-	-	137	(55.7%)	148	(27.7%)	< 0.001
Polypharmacy [*N*(%)]	137	(48.1%)	109	(22.0%)	< 0.001	-	-	-
NIHSS (Median[*IQR*])	4	[2;11]	3	[1;9]	0.032	4	[2;12]	3	[1;9]	0.018
Living at home^*^ [*N*(%)]	187	(65.6%)	430	(86.7%)	< 0.001	179	(72.8%)	438	(81.9%)	0.005
Independence† [*N*(%)]	231	(81.1%)	471	(95.0%)	< 0.001	214	(87%)	488	(91.2%)	0.091
Atrial Fibrillation [*N*(%)]	91	(38.6%)	148	(27.2%)	0.002	97	(39.4%)	142	(26.5%)	< 0.001
Hypertension [*N*(%)]	153	(65.1%)	313	(58.1%)	0.079	180	(73.5%)	286	(54.1%)	< 0.001
Hyperlipidemia [*N*(%)]	34	(14.9%)	57	(10.8%)	0.145	36	(15.3%)	55	(10.6%)	0.085
Deceased by 90 day follow up	42/236	(17.8%)	29/545	(5.3%)	< 0.001	34/246	(13.8%)	37/535	(6.9%)	0.003
Global physical health T-Score (Mean ±*SD*)	40.2	[±9.54]	44.9	[±9.9]	< 0.001	41	[±8.92]	44.8	[±10.2]	< 0.001
Global mental health T-Score (Mean ±*SD*)	41	[±7.51]	44.3	[±8.99]	0.001	41.2	[±7.69]	44.3	[±9.01]	0.001
PROMIS-10 General Health (Mean ±*SD*)	2.42	[±0.86]	2.78	[±0.98]	< 0.001	2.43	[±0.77]	2.79	[±0.96]	< 0.001
PROMIS-10 General Social (Mean ±*SD*)	3.12	[±1.09]	3.63	[±1.2]	< 0.001	3.21	[±1.19]	3.62	[±1.18]	0.002

* Without external support or care † in dressing, mobility and toileting before index stroke.

### Influence of CCI score and polypharmacy on self-reported quality of life

We first performed univariate analyses with Global Physical Health T-Score and Global Mental Health T-Score as outcome measures (see Supplementary Table 3). We then fitted linear regression models including parameters which significantly predicted outcomes in univariate analyses with *p* < 0.05. We excluded Hypertension due to high collinearity with polypharmacy. In multivariate regression analyses (see Table 3) a CCI Score ≥2 was independently associated with worse self-reported outcome score in both physical (ß = −0.142, p 0.002) and mental health (ß = −0.102, *p* 0.034), while polypharmacy was not statistically significantly associated with either.

**Table 3:** Association between predictors and outcomes in multivariable analyses.

	**PROMIS-10 Global Physical Health T-Score** ***R***^**2**^ = **0.23**, ***R***^**2**^ **adjusted** = **0.21**, ***F***_**(8, 389)**_ = **14.53, p-Value of the model<0.001**					**PROMIS-10 Global Mental Health T-Score** ***R***^**2**^ = **0.17**, ***R***^**2**^ **adjusted** = **0.15**, ***F***_**(8, 388)**_ = **9.95**, ***p*****-Value of the model<0.001**					**VIF‡ **	**Tolerance**
		**95% CI**					**95% CI**					
**Predictor**	**EST**	**Lower**	**Upper**	(β)^*^	***p*** **-value**	**EST**	**Lower**	**Upper**	(β)^*^	***p*** **-value**		
Intercept	54.9	49.8	60.0	/	< .001	50.6	36.8	49.9	/	< 0.000	/	/
Female sex	−3.09	−4.91	−1.28	−0.154	< .001	−2.97	−4.63	−1.32	−0.169	< 0.001	1.08	0.93
Age	−0.06	−0.14	0.02	−0.076	0.126	−0.02	−0.09	0.05	−0.029	0.573	1.2	0.83
CCI Score ≥2	−3.34	−5.47	−1.2	−0.142	0.002	−2.1	−4.04	−0.15	−0.102	0.035	1.08	0.92
Polypharmacy	−1.71	−3.83	0.41	−0.076	0.113	−1.60	−3.52	0.31	−0.082	0.101	1.13	0.89
NIHSS	−0.33	−0.5	−0.17	−0.188	< 0.001	−0.19	−0.33	−0.04	−0.119	0.014	1.07	0.93
Dependency†	−5.1	−9.34	−0.86	−0.109	0.018	−4.27	−8.25	−0.3	−0.102	0.035	1.08	0.94
Atrial Fibrillation	−3.45	−5.52	−1.39	−0.156	0.001	−2.31	−4.2	−0.43	−0.12	0.016	1.14	0.88

* Standardized coefficient (β).

†Dependency concerning toileting, dressing and mobility.

‡Variance Inflation Factor to test for multicollinearity within the model.

To test for multicollinearity within the model, we calculated the tolerance and variance inflation factor for the regression model. The tolerance was >0.1 for all predictors, indicating a low likelihood of multicollinearity within the model. With the variance inflation factor smaller than two for all predictors, no severe multicollinearity was detected (see Table 3). Sensitivity analysis including patients with missing outcome values after multiple imputation confirmed comorbidity as a predictor of worse physical and mental health at 90 days after stroke (see Supplementary Table 4). With regard to other predictors, pre-existing hypertension was no longer a significant predictor of worse physical or mental health while age was significant for predicting worse physical health.

## Discussion

In this secondary *post-hoc* analysis of the EPOS study, we evaluated the association of pre-existing comorbidity burden and polypharmacy with self-reported measures of physical and mental health 90 days after acute ischemic stroke. Our study was motivated by the increasing prevalence of high comorbidity burden and polypharmacy and lack of data on their impact on HrQoL. As its main finding our study yielded that comorbidity burden was both frequent in stroke patients, and an independent predictor of worse physical and mental health. Polypharmacy had a high prevalence in the cohort studied but showed no significant association with the evaluated outcome measures. Our data thus suggest that comorbidity burden as evaluated by the CCI Score is a meaningful measure for identifying a population at risk for suffering of detrimental effects on physical and mental health after AIS.

A high comorbidity burden in patients with AIS is a growing challenge in clinical practice and patient management. Previous studies reported 19% to 64% of stroke patients presenting with a CCI Score ≥2 (27, 28). In an aging society, the comorbidity burden of patients is likely to increase further. With 36.5% the number of patients in this study having a CCI Score ≥2 was in line with findings from previous studies. Patients with higher comorbidity burden were older, more often had polypharmacy, less often lived at home or independent and had more severe stroke symptoms on admission. At 90 days after stroke, they had higher mortality and worse outcomes in all measures of self-reported HrQoL. These results identify stroke patients with a high comorbidity burden as a vulnerable subgroup requiring specific attention to avoid detrimental outcome. The negative influence of a high comorbidity burden on functional outcomes after stroke is well-studied (9, 10). Previous studies trying to determine influencing factors on HrQoL after stroke mostly did not account for comorbidity burden, but showed factors influencing HrQoL to be similar to those influencing functional outcome (2, 3, 5). In a large Korean study of 2,289 patients with first-ever stroke, comorbidity burden measured by the condition- and age-adjusted CCI score was associated with worse QoL 6 months after stroke in univariate analysis, but not in multivariate analysis when further demographic and clinical parameters were studied (29). In another cohort study, higher CCI values were associated with worse scores in the physical component summary of the Short Form 12 Health Survey 6 months after stroke (30). Our findings add to this and suggest the use of a dichotomized CCI with a cut-off ≥2 as a valuable indicator for identifying patients at risk for worse mental and physical HrQoL after stroke.

Polypharmacy was present in 31.5% of our patients, which is in the upper range of reported polypharmacy rates between 10 and 30% in previous studies (28, 31–33). While in univariate analysis, polypharmacy had a negative association with both outcome measures, this association was no longer present in multivariate analysis. A *post-hoc* analysis of “WAKE UP” showed an association of polypharmacy with worse functional outcome independent of CCI (28). The authors discussed the possible attribution of their findings to a selection effect caused by the strict in- and exclusion criteria in this randomized controlled trial. Our data from an unselected cohort of stroke patients from clinical practice suggest that polypharmacy is likely to play a role in predicting worse HrQoL as an indicator of a higher comorbidity burden. Antihypertensives, for example, were the most common class of medication in our population, contributing heavily to polypharmacy, and hypertension remained a significant predictor of worse HrQoL in multivariate analysis. With regard to further predictors of HrQoL after stroke, our results were largely in line with findings of previous studies. The index stroke severity in our patient cohort was low to moderate with a median NIHSS of 4. Consistent with previous findings, severity of stroke symptoms reflected by higher initial NIHSS scores had a negative impact on HrQoL (1, 2, 5). Female sex also predicted worse HrQoL in both domains, as was shown in previous studies (4, 34). This association may be influenced by women's advanced age, greater stroke severity and poorer health at time of stroke and worse access to health care due in part to social isolation (4). Modifiable sex specific risk factors contributing to worse outcome are needed to reduce sex disparities in outcome. Pre-morbid functional level is known to influence HrQoL after stroke (1), this was reproducible in our cohort. Patients requiring help in activities of daily living pre-stroke, i.e., help in domains of toileting, dressing or mobility, had worse HrQoL 3 months after stroke. Atrial fibrillation is known to be independently associated with worse HrQoL (35). As atrial fibrillation is also a major risk factor for recurrent stroke, this identifies stroke patients with atrial fibrillation as an especially vulnerable population both with regards to HrQoL and recurrent events.

Higher age only had an influence on HrQoL in univariate analysis that didn‘t persist in multivariate analysis. This could be attributed to an increase in comorbidity burden with age, causing age to lose its effect after correcting for high comorbidity burden. Furthermore, it might be explained by the fact that baseline age in this cohort was above retirement age, relieving patients from the burden of socio-economic pressures connected to earning a living. Limitations to this study include a relatively high drop-out rate for follow-up data with significant differences between completers and non-completers. This may have biased our findings and limit the generalizability of our results. Regression analyses with the intention-to-treat population however, confirmed the association of comorbidity burden with worse functional health status. Moreover, our analysis was not powered to study specific effects of individual comorbidities comprised in the CCI or individual drugs or medication classes in detail.

## Conclusions

To summarize, both a CCI Score ≥2 and polypharmacy had a high prevalence of more than one third in our cohort of patients with AIS from clinical practice. A higher comorbidity burden was an independent predictor of worse scores of self-reported physical health and mental health, while polypharmacy was not. These results point toward the importance of comorbidity for HrQoL after stroke and may help in identifying patients at risk for poor outcome after stroke.

## Data availability statement

The raw data supporting the conclusions of this article will be made available by the authors, without undue reservation.

## Ethics statement

The studies involving human participants were reviewed and approved by Ethics Committee of the Hamburg chamber of physicians. The patients/participants provided their written informed consent to participate in this study.

## Author contributions

MHe and GT conceived and designed the study, conducted the statistical analysis, interpreted the data, and wrote the first draft of the manuscript. MF, MJ, EB, IL, and MHä acquired data. LL, DR, LK, and CG acquired data and critically revised the manuscript. All authors contributed to the article and approved the submitted version.

## Funding

The EPOS study on which this secondary analysis is based is funded by the Innovation Fund of the German Federal Joint Committee with grant number 01VSF16023. The German Federal Joint Committee reviewed and approved the study design during the grant application process. It had no role in the conduct of the study or publication process.

## Conflict of interest

Author CG reports personal fees from Amgen, Bayer Vital, Bristol-Myers Squibb, Boehringer Ingelheim, Sanofi Aventis, Abbott, and Prediction Biosciences outside the submitted work. Author GT reports receiving consulting fees from Acandis, grant support, and lecture fees from Bayer, Boehringer Ingelheim, Bristol-Myers Squibb/Pfizer and Daiichi Sankyo, and consulting fees and lecture fees from Stryker outside the submitted work. The remaining authors declare that the research was conducted in the absence of any commercial or financial relationships that could be construed as a potential conflict of interest.

## Publisher's note

All claims expressed in this article are solely those of the authors and do not necessarily represent those of their affiliated organizations, or those of the publisher, the editors and the reviewers. Any product that may be evaluated in this article, or claim that may be made by its manufacturer, is not guaranteed or endorsed by the publisher.

## Abbreviations

AIS: acute ischemic stroke
CCI: Charlson Comorbidity Index
HrQoL: health-related quality of life
NIHSS: National Institutes of Health Stroke Scale
PROMs: patient-reported outcome measures
PROMIS-10: Patient-reported Outcomes Measurement Information System 10-Question Short-Form.
