# Supplementary material for: Higher comorbidity burden is associated with lower self-reported quality of life after stroke
Source: Front Neurol. 2022 Nov 10;13:1023271. doi: 10.3389/fneur.2022.1023271 (PMC9685789; doi:10.3389/fneur.2022.1023271)
Supplement: Supplementary file 1 [file Data_Sheet_1.pdf]

Supplementary Material

1 Supplementary Tables

Supplementary Table 1. Patient characteristics and PROMIS-10 scores at 90 day follow up

**Variables**		**Overall *N****
Deceased by 90 day follow up (*N*(%))	71 (14.76%)	481
Living at home† (*N*(%))	308 (75.3%)	409
Independence‡ (*N*(%))	335 (82.1%)	408
Global Physical Health T-Score (Mean±*SD*)	43.8 [±10.0]	401
Global Mental Health T-Score (Mean± *SD*)	43.5 [±8.76]	400
PROMIS-10 General Health (Mean± *SD*)	2.69 (±0.92)	408
PROMIS-10 General Social (Mean± *SD*)	3.51 (±1.19)	407

* in case of missing data, *N* of available for data is displayed
† without external support or care
‡ in dressing, mobility and toileting before index stroke

Supplementary Table 2. Patients lost to follow up

**Variables**	**Non-Completers *N* = 301**		**Completers *N* = 409**		***p*-Value**
Female (*N*(%))	144	(47.8%)	194	(47.4%)	.975
Age (Mean±*SD*)	74	(±13)	72.5	(±13)	.118
CCI Score ≥2	97	(32.2%)	97	(23.7%)	.015
Polypharmacy (*N*(%))	99	(32.9%)	113	(27.6%)	.152
NIHSS (Median [*IQR*])	4	[2;12]	3	[1;6]	<.001
Living at home* (*N*(%))	227	(73.5%)	361	(90.0%)	<.001
Independence† (*N*(%))	265	(88%)	389	(95.1%)	.001
Atrial Fibrillation (*N*(%))	91	(30.2%)	118	(28.9%)	.752
Hypertension *N*(%))	170	(57%)	256	(63.1%)	.125
Hyperlipidemia (*N*(%))	28	(9.6%)	55	(14%)	.100

* without external support or care
† in dressing, mobility and toileting before index stroke

Supplementary Table 3. Association between predictors and outcomes in univariable analyses

	PROMIS-10 Global Physical Health T-Score				PROMIS-10 Global Mental Health T-Score			
		95% CI				95% CI		
**Predictor**	**EST**	**Lower**	**Upper**	***p*-Value**	**EST**	**Lower**	**Upper**	***p*-Value**
Female Sex	-4.86	-6.77	-2.95	<.001	-4.14	-5.82	-2.46	<.001
Age	-0.19	-0.27	-0.12	<.001	-0.13	-0.20	-0.07	<.001
CCI Score ≥2	-4.69	-6.96	-2.43	<.001	-3.25	-5.25	-1.25	.002
Polypharmacy	-3.76	-5.94	-1.57	<.001	-3.10	-5.00	-1.19	.002
NIHSS	-0.46	-0.63	-0.29	<.001	-0.29	-0.45	-0.14	<.001
Dependency*****	-9.55	-14.1	-5.02	<.001	-7.71	-11.8	-3.62	<.001
Atrial Fibrillation	-5.94	-8.04	-3.84	<.001	-4.04	-5.91	-2.17	<.001
Hypertension	-4.14	-6.14	-2.14	<.001	-3.87	-5.62	-2.12	<.001
Hyperlipidemia	-0.40	-3.37	2.56	.789	-0.21	-2.80	2.38	.872

*in dressing, mobility and toileting before index stroke

Supplementary Table 4. Association between predictors and outcomes in multivariable analyses using multiple imputation

	PROMIS-10 Global Physical Health T-Score					PROMIS-10 Global Mental Health T-Score				
		95% CI					95% CI			
**Predictor**	**EST**	**Lower**	**Upper**	**(β)***	***p*-Value**	**EST**	**Lower**	**Upper**	**(β)***	***p*-Value**
Intercept	48.6	41.6	55.6	/	<.001	43.72	36.22	51.21	/	<.000
Female Sex	-3.07	-4.99	-1.15	0.403	.002	-3.12	-4.76	-1.48	0.258	<.001
Age	-0.10	-0.17	-0.02	0.001	.013	-0.05	-0.12	0.03	0.001	.215
CCI Score ≥2	-3.89	-6.07	-1.72	0.545	<.001	-2.31	-4.17	-0.46	0.353	.015
Polypharmacy	-1.9	-3.99	0.2	0.483	.075	-1.56	-3.56	0.45	0.501	.127
NIHSS	-0.36	-0.53	-0.18	0.005	<.001	-0.21	-0.37	-0.06	0.004	.007
Dependency†	-4.71	-8.36	-1.09	1.72	.012	-4.89	-8.92	-0.87	2.625	.018
Atrial Fibrillation	-2.87	-4.96	-0.78	0.431	.431	-2.01	-3.56	-0.45	0.501	.036

* Standardized coefficient (β)
† Dependency concerning toileting, dressing and mobility

2 Supplementary Figures

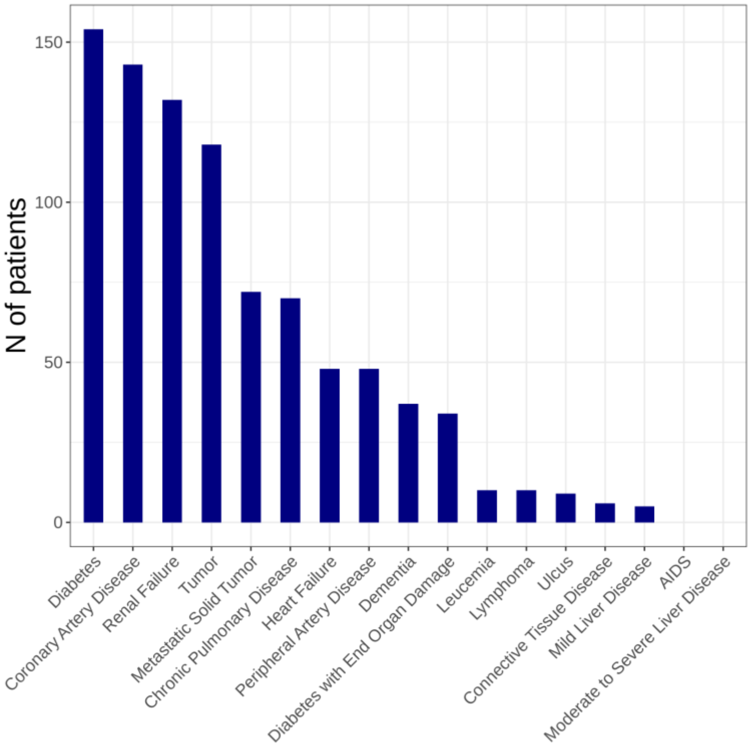

Supplementary Figure 1 illustrates the proportion of patients suffering of each condition listed as an item in the CCI at time of index stroke.

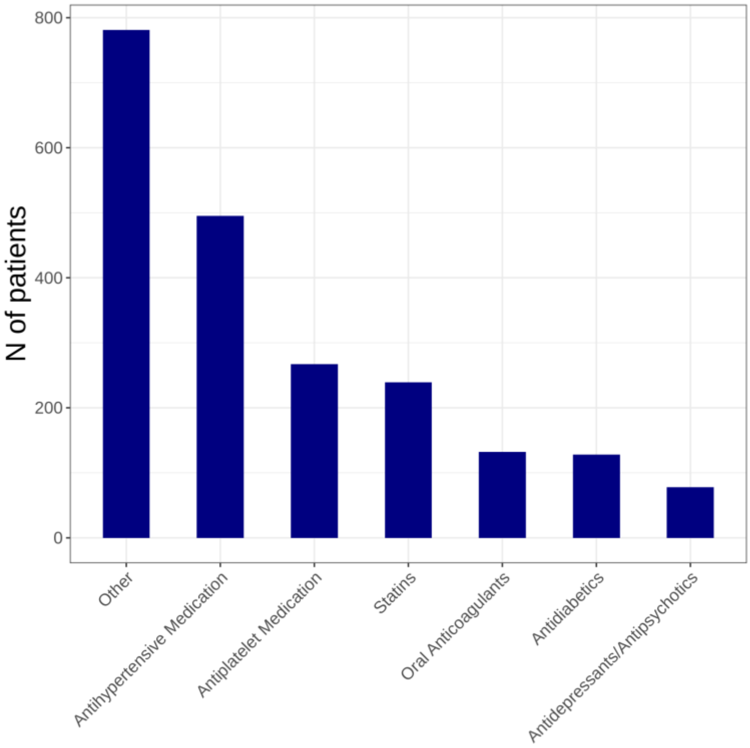

Supplementary Figure 2 illustrates the number of patients taking at least one drug of different medication classes at time of index stroke.
